# Benchmarking machine-readable vectors of chemical reactions on computed activation barriers†

**DOI:** 10.1039/d3dd00175j

**Published:** 2024-03-07

**Authors:** Puck van Gerwen, Ksenia R. Briling, Yannick Calvino Alonso, Malte Franke, Clemence Corminboeuf

**Affiliations:** a Laboratory for Computational Molecular Design, Institute of Chemical Sciences and Engineering, École Polytechnique Fédérale de Lausanne 1015 Lausanne Switzerland clemence.corminboeuf@epfl.ch; b National Center for Competence in Research-Catalysis (NCCR-Catalysis), École Polytechnique Fédérale de Lausanne 1015 Lausanne Switzerland

## Abstract

In recent years, there has been a surge of interest in predicting computed activation barriers, to enable the acceleration of the automated exploration of reaction networks. Consequently, various predictive approaches have emerged, ranging from graph-based models to methods based on the three-dimensional structure of reactants and products. In tandem, many representations have been developed to predict experimental targets, which may hold promise for barrier prediction as well. Here, we bring together all of these efforts and benchmark various methods (Morgan fingerprints, the DRFP, the CGR representation-based Chemprop, SLATM_*d*_, *B*^2^*R*_*l*_^2^, EquiReact and language model BERT + RXNFP) for the prediction of computed activation barriers on three diverse datasets.

## Abstract 2

We benchmark various methods for the prediction of computed activation barriers on three diverse datasets.

## Graphical abstract

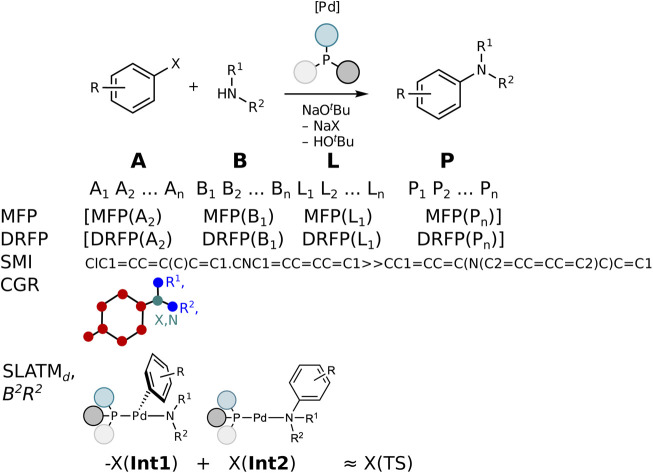

## 1 Introduction

The activation barrier is a fundamental quantity required to understand elementary reaction steps, allowing the estimation of reaction rates and determining dominant reaction pathways.^1–5^ Obtaining an accurate Transition State (TS) structure (and therefore, its energy) remains a major computational bottleneck in reaction exploration tasks.^2,4,6–10^ Machine learning of activation barriers offers a cheaper alternative than direct computation. Consequently, there has been a recent flurry of works aiming at accurately predicting them.^11–29^ These models have featurized reactions using the 2D graph of reactants and products,^13–16^ Physical Organic (PO) descriptors derived from computations on reactant and product molecules,^17–20^ or 3D structure of reactants and/or products.^21–29^

Of the 2D-graph-based models, the frontrunner is the “Condensed Graph of Reaction” (CGR)^13,14^ used to represent reactions in the Chemprop^30,31^ model, which is constructed by exploiting atom-mapped reaction SMILES such that a node in the Graph Neural Network (GNN) describes an atomic centre that is transformed during a reaction. Note that atom-mapping remains a challenge in digital chemistry^32,33^ despite the progress made over decades of effort.^34–44^ State-of-the-art atom-mapping tools either enumerate a subset of known chemical transformations and try to identify them in the reaction of interest,^45^ or “template-free” models attempt to extract chemical transformation rules from data.^46^ Both approaches are restricted to commonly-occurring organic chemistry: the former requiring the enumeration of reaction rules, and the latter trained on organic chemistry from patent data.^47^ Both codes pose practical problems, too, being closed-source. In many cases, it is still preferable to atom-map manually, as was done by Heid *et al.*^13^ for specific reaction classes. Atom-mapping by hand requires knowledge of the reaction mechanism for every step in a multi-step process, which is not available for most new chemistry. Given a dataset is correctly atom-mapped however, the CGR representation in Chemprop (hereafter referred to as Chemprop) is a promising approach to encode reactions.^13,14,31^ Chemprop has been used to predict computed reaction barriers^48–51^ as well as computed reaction energies,^52^ reaction rate constants,^53^ experimental activation energies,^54^ yields^55^ and reaction classes.^56,57^

“Physical Organic” (PO) descriptors are based on the properties of molecules involved in the reaction, typically using quantum-chemical computations.^18,54,58–67^ The computed properties can be broadly divided into steric and electronic parameters.^54,58,63,64,68^ Steric properties include the molecular volume and surface area or Sterimol parameters,^69,70^ or in the case of ligands, buried volume^63^ or Tolman cone angles.^71^ Electronic descriptors include frontier molecular orbital energies, reactivity indices from conceptual DFT,^72^ natural bond orbital (NBO)-derived descriptors, atomic charges, and NMR chemical shifts.^64^ There are also conformation-specific descriptors such as the Average Steric Occupancy (ASO)^60^ and Molecular Field Analysis (MFA)-based descriptors.^73^ Typically, PO descriptors that are relevant for a specific reaction are chosen by an expert. However, databases of descriptors are being pre-computed and made publicly available,^74^ and automated workflows developed,^75–77^ that alleviate the need of an expert to construct these representations.^65^ So-called QM-augmented GNNs^77–79^ combine atom-mapped graph-based models (specifically, Weisfeiler–Lehman Networks (WLNs)^80^) and PO descriptors. QM-GNNs^78,79^ as well as PO descriptors combined with simpler (often linear) models^17,18,20,81^ have been used to predict activation barriers. PO-based models have also been used to predict experimental targets such as yield^58,59^ and e.e.^60,62,82,83^

**Fig. 1:**
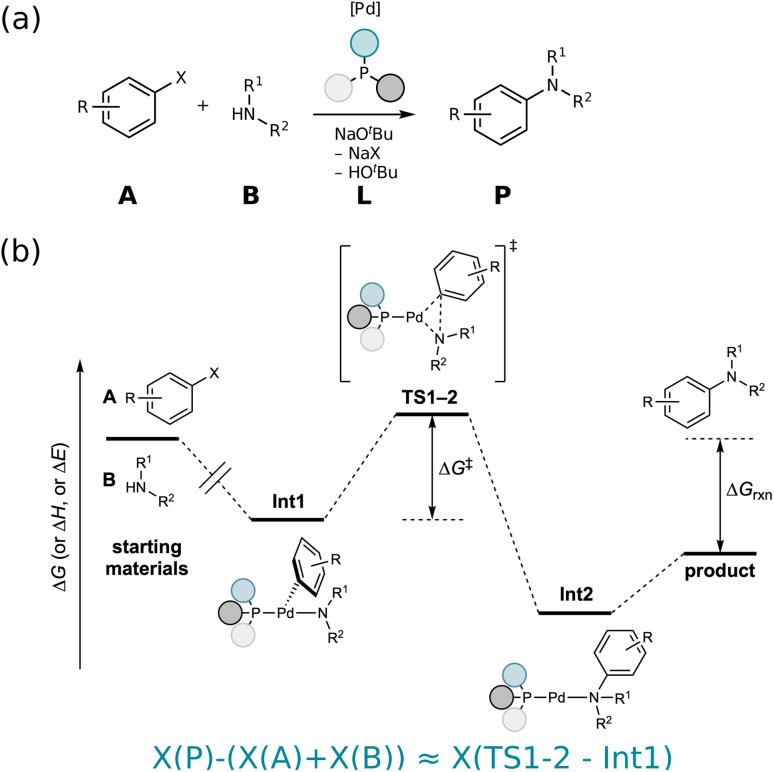
(a) An example Buchwald–Hartwig amination is illustrated with starting materials A and B, catalyzed by a Pd-based catalyst with phosphene ligands L resulting in product P. (b) Thermochemistry-inspired representations aim to predict the activation barrier Δ*G*^‡^, which is a function of the TS1-2 and Int1 energies, using starting materials and products (or intermediates Int1 and Int2, if this mechanistic information is available).

3D-structure-based models can be broadly separated into two categories: (i) those that predict the TS structure, by virtue of Generative Adversarial Networks (GANs),^24^ GNNs,^23^ Reinforcement Learning (RL)^22^ or diffusion models,^21,84^ or (ii) those that directly predict the activation barrier,^13,14,25,27–29^ which will be our focus here. As illustrated in Fig. 1, such 3D-structure based reaction representations can be thought of as “thermochemistry-inspired”, using an interpolation between reactants' and products' geometries as a proxy^85^ for the reactants and TS, that allows for a mapping to the activation barrier. Examples are SLATM_*d*_,^28,29^ constructed from molecular representations^86^ of reactants and products and *B*^2^*R*^2^,^29^ a dedicated reaction representation. Molecular variants of these representations^86–91^ are often referred to as “physics-based”,^92–94^ taking as input molecules' atom types and coordinates (and in some cases charge and spin information^95,96^) thereby mimicking the role of the Hamiltonian in the Schrödinger equation to solve for molecular properties.

In a similar spirit, physics-based deep learning models^97–106^ learn their own representations from the structural input data (*i.e.* nuclear charges and coordinates) and encode known physical priors, such as symmetries, into the network architecture. While these have been demonstrated to obtain impressive out-of-sample accuracies for molecular properties on countless occasions,^97–106^ they have only recently been introduced to predict reaction properties.^14,107^ EquiReact,^107^ an Equivariant Neural Network (ENN) that uses 3D information from reactants and products, as well as optionally atom-mapping information, has been shown to exhibit competitive performance on datasets of reaction barriers.

In tandem to the work on activation barrier prediction, there has been a broader effort to predict experimental targets,^11,58,60,108–111^ including reaction class,^56^ synthetic routes^46,112–115^ and experimental outcomes (yield,^58,59,109,110^ activation energy^54^ and enantioselectivity e.e.^60,62^). The workhorse reaction representation in this domain is a combination of Morgan FingerPrints (MFPs)^116^ of reaction components.^117–119^ MFPs identify the presence of circular substructures in a 2D description of molecules. Alternatively, reactions can be represented as strings in the form of reaction SMILES. Powerful transformer models^120,121^ developed for language translation can then be applied to text-based descriptions of reactions for classification or regression tasks.^46,56,109,112^ Such models were originally explored in chemistry with synthesis planning tasks in mind.^46,112^ Since then, the RXNFP^56^ has been used to predict reaction classes^56^ and yields^109^ as well. The Differential Reaction FingerPrint (DRFP), which takes a symmetric difference between substructures identified in the reactants and products to generate a fixed-size vector per reaction, has been illustrated to be competitive with the RXNFP for related yield and reaction class prediction tasks.^122^

Here, we benchmark different reaction representations, whether previously used to predict activation barriers (the graph-based Chemprop,^13,31^ physics-based SLATM_*d*_ and *B*^2^*R*_*l*_^2^,^29^ EquiReact^107^) or not (MFPs,^116^ DRFP,^122^ and RXNFP^56,109^) for the prediction of activation barriers across several datasets: from the general-scope GDB7-22-TS^123^ to a single-reaction class dataset Cyclo-23-TS^51^ to a specific dataset, the Proparg-21-TS.^28,124^ In the process, we discover what information needs to be captured in the reaction representation for accurate activation barrier prediction.

## 2 Computational details

### 2.1 Representations and ML models

We study a variety of reaction representations: the 2D-structure based MFP^116^ and DRFP,^122^ the 2D-graph based Chemprop,^13,31^ RXNFP^56^ trained using the BERT language model,^120^ 3D-structure based thermochemistry-inspired representations SLATM_*d*_ and *B*^2^*R*_*l*_^2^,^29^ and ENN EquiReact.^107^ Note that we exclude PO descriptors because of the computational cost associated with generating these representations for the larger databases studied in this work.

#### 2.1.1 MFP and DRFP

The input to these models is the SMILES strings of reactants and products, or the reaction SMILES. A difference of Morgan FingerPrints of reactants and products (MFP) was generated using RDKit version 2023.3.3.^125^ The Differential Reaction FingerPrint (DRFP) was generated using the drfp version 0.3.6.^122^ A fingerprint size of 1024 was used throughout. Fingerprints are combined with random forest (RF) models as implemented in sklearn^126^ version 1.3.1, as these models are naturally suited to the binary features in MFP and DRFP fingerprints. Correspondingly, MFP/DRFP have often been combined with RF or gradient boosting^127^ models in previous publications for best results.^13,31,58,122,128^

#### 2.1.2 Chemprop

The Condensed Graph of Reaction (CGR)^13^ is built from atom-mapped SMILES strings of reactants and products, which is then passed through the directed message-passing neural network Chemprop^30^ (version 1.6.1, using RDKit^125^ version 2023.9.4).

In order to assess the sensitivity of the trained models to the quality of the atom-mapping, we tested three versions of each Chemprop model: (i) with “true” atom-mapping (“Chemprop True”), as provided by the authors of the datasets (typically obtained using the transition state structure, or using known reaction rules); (ii) with “automatic” atom-mapping, performed by the open-source tool RXNMapper^129^ (“Chemprop RXNMapper”); and (iii) with no atom-mapping, which removes the atom-mapping indices from reactants and products (“Chemprop None”). The latter option evaluates the efficacy of the graph-based models without atom-mapping information. We note that this is different to how the “no-mapping” model for Chemprop was run for a previous publication,^130^ where the mappings of product atoms were randomly shuffled with respect to reactant atoms. In the case of no maps, Chemprop interprets the reactants' and products' graphs as separate entities. For an unmapped reaction A + B → C, it extends the reactants' and products' graphs with “ghost” graphs shadowing the missing counterparts producing the disconnected reaction graph (A.B.0^C^) → (0^A^.0^B^.C). The condensed graph, being (with default settings) a difference between products and reactants, reads (−A.−B.C). This leads to a ∼1 kcal mol^−1^ improvement compared to “no mapping” results with random maps.^130^

Where available (for the GDB7-22-TS and Proparg-21-TS sets with “True” maps), we used explicit hydrogen atoms in the Chemprop model. Otherwise, the Hs are implicit.

We also note that the version of RXNMapper used here (0.3.0) runs successfully on all datasets studied, while the previous version (0.2.9) failed on some datasets in a previous publication.^130^

#### 2.1.3 Language models

Language models are built from reaction SMILES. Input data is augmented using SMILES randomization:^131,132^ first, SMILES strings of reactants and products are de-canonicalized. Then, atoms of each SMILES string are renumbered by rotation of their index. For each renumbering, a grammatically correct SMILES is constructed. Duplicate SMILES are removed after the randomization procedure. Using a randomization factor of 10, this effectively multiplies the training and test set sizes by 10 for each dataset. Since most of our reactions consist of a single reactant and product, we did not employ molecule permutations, also illustrated to be an effective data augmentation strategy.^132^

We use a BERT model^120^ pre-trained on a reaction MLM task from the rxnfp library.^133^ We fine-tune the model on the training data. 10× data augmentation was used (see the ESI† for a comparison of models with and without data augmentation).

#### 2.1.4 Thermochemistry-inspired representations

In Fig. 1a, an example Buchwald–Hartwig amination is illustrated with starting materials A and B, catalyzed by a Pd-based catalyst with phosphene ligands L resulting in product P. The reaction mechanism is shown in Fig. 1b. While the activation barrier Δ*G*^‡^ can be obtained from the energies of the Transition State (TS1-2) and the preceding state (Int1), determining the structure of the TS is computationally expensive. Likewise, an ML model to predict Δ*G*^‡^ would be most accurate if the geometry of the TS was used to construct the representation. However, if the geometry is known, the energy is known, making the ML model redundant. Thermochemistry-inspired representations, that instead use 3D structures of reactants and products, have therefore been developed.^28,29^

Depending on the mechanistic information available, “reactants” can be either starting materials or the intermediate preceding the relevant TS. Similarly, “products” may be an intermediate following the TS or the final product. For uncatalyzed reactions, using starting materials and products only is sufficient. For catalyzed reactions however, both the catalyst/ligand and substrates must be encoded. Representations built from intermediates preceding and following the TS have the advantage of naturally encoding the structure of the catalyst.

A previous benchmarking study^29^ identified the best-performing thermochemistry-inspired representation as the difference in SLATM vectors^86^ of reactants and products (SLATM_*d*_). The molecular SLATM representation is built from increasing orders of potential terms that describe interactions between atoms in a molecule.^86^ The one-, two- and three-body terms are concatenated to form the eventual molecular vector. The *B*^2^*R*^2^ family of representations^29^ are constructed in a similar way, except using different potential functions and being truncated at two-body terms. The *B*^2^*R*_*l*_^2^ representation employed here combines the appropriate features into element-wise “bags” depending on the identity of the element *I* in each pairwise interaction between atoms *I*, *J*. SLATM_*d*_ instead bags pairwise interactions into pairwise bags, as well as three-body interactions into bags corresponding to the identity of the three involved elements.

Molecular SLATM vectors were generated using the qml python package^134^ before being combined to form the reaction version SLATM_*d*_. The *B*^2^*R*_*l*_^2^ representation is generated from the github repository,^135^ using default parameters. These representations are combined with Kernel Ridge Regression (KRR) models, as has been the standard for the molecular representations since their initial development.^89,128,136–141^ So-called “physics-based” representations are typically high dimensional, continuous, and used in a low-data regime. Kernel methods are then well-suited to these, allowing for meaningful interpretation of the similarity kernel, finding trends in high dimensions and with little data.^92–94^

#### 2.1.5 EquiReact

EquiReact^107^ builds on the thermochemistry-inspired representations, taking the same structures as input to the model. However, the representation is learned end-to-end as part of the training process. The model consists of independent equivariant channels for reactant and product molecules, followed by different possible combination modes to obtain a latent reaction representation (which can take into account atom-mapping information, mimic atom-maps with cross-attention, or use simple arithmetic operations like the difference between reactants' and products' representations). The latent representation is provided as input to an MLP to predict the reaction barrier. Here, we take the model either using atom mapping or not resulting in the best performance in each case (GDB7-22-TS with atom-maps, the other models without). The set of hyperparameters for these models is given in the ESI.†

As in the original work,^107^ we run EquiReact without explicit Hs, as it has been shown that there is no consistent improvement in performance when including Hs, and the models become significantly more expensive to train.

### 2.2 Data splits, hyperparameters and performance metrics

All datasets are split into 10 random 80% train/10% validation/10% test splits. For all models, performance is reported as mean absolute error (MAE) on the test set, averaged over the 10 folds.

In the case of the EquiReact model, we use the previously published hyperparameters,^107^ which correspond to those optimised on the first data split for each of the three datasets. These are repeated in the ESI† of this paper.

For all other models, hyperparameters are tuned for the first data split and then used for all other splits. The space of hyperparameters tested, as well as the optimal parameters obtained, can be found in the ESI.† In the case of a large number of parameter combinations (the RF models and Chemprop), the parameters were optimized using Bayesian optimization with hyperopt version 0.2.7.^142–144^ For the RF models, we optimized the maximum depth of the decision trees, the number of decision trees, the maximum number of features used to split each tree, the minimum number samples per split, minimum number of samples per leaf, and whether to bootstrap the models. A maximum of 100 combinations of hyperparameters were tested for each model.

For Chemprop, the hyperparameter search using hyperopt was modified from the chemprop codebase,^145^ version 1.6.1, to evaluate the hyperparameters on the first cross-validation fold only (the default behaviour finding the best hyperparameters over *k* folds). The modified code can be found in forked version^146^ of the original repository. As per the default in the original codebase, the hyperparameter search optimizes the hidden size, depth (number of message passing iterations), dropout probability and number of feed-forward layers after message passing. For the hyperparameter search, we train the models for 100 epochs (50 for the GDB7-22-TS set), rather than the 300 epochs used to train and test the model performance. A maximum of 100 (50 for the GDB7-22-TS set) combinations of hyperparameters were tested for each model.

For the kernel models with relatively few hyperparameters, a grid search was used to optimize the kernel width and regularization parameter. For RXNFP, following the suggestions in the documentation,^147^ we optimize the learning rate and dropout probability parameters using a grid search. We use a batch size of 32 and train for 10 epochs.

### 2.3 Datasets of reaction barriers

In order to compare reaction representations built from either reaction SMILES or three-dimensional structure, we include two recently published datasets of activation barriers that provide both input formats: the GDB7-22-TS^123^ and Cyclo-23-TS.^51^ Several models^31,79,107^ have already been tested on these sets, which allows for an interesting broader comparison across different reaction fingerprints. We also include the Proparg-21-TS set of reaction barriers,^28,124^ which provides only three-dimensional structure as the input format. In order to allow comparison to other methods we convert these to reaction SMILES (*vide supra*).

**Fig. 2:**
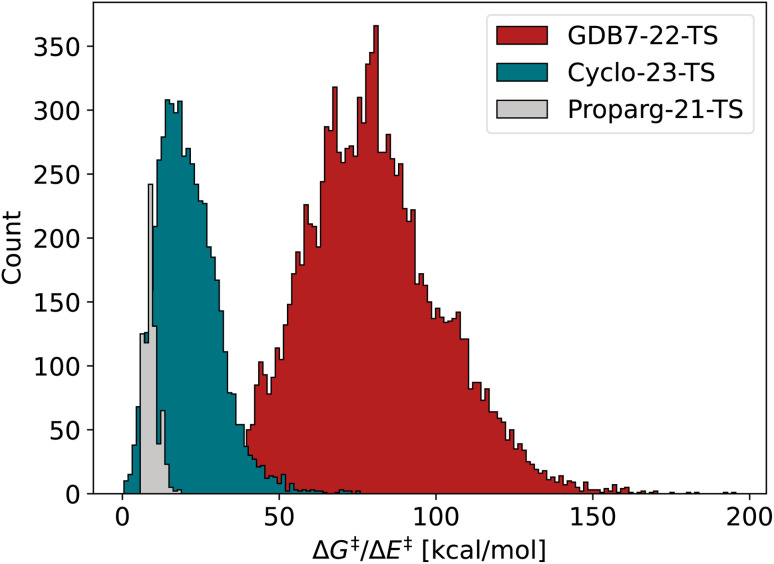
Distribution of barriers Δ*G*^‡^ or Δ*E*^‡^ for the three datasets GDB7-22-TS, Cyclo-23-TS and Proparg-21-TS.

The three datasets are diverse in their respective challenges. Fig. 2 illustrates the spread in the barrier (Δ*G*^‡^ or Δ*E*^‡^) for the sets, highlighting their difference. Each is described in detail below.

#### 2.3.1 GDB7-22-TS

The GDB7-22-TS dataset^123^ consists of close to 12 000 diverse un-catalyzed organic reactions automatically constructed from the GDB7 dataset^148–150^ using the growing string method^151^ along with corresponding energy barriers (Δ*E*^‡^) computed at the CCSD(T)-F12a/cc-pVDZ-F12//ωB97X-D3/def2-TZVP level. These molecules have a maximum of 7 heavy atoms and a maximum of 23 atoms. The input structures to the ML models are optimized reactants (starting materials) and products. This is an updated version of the GDB7-20-TS set^48^ used in previous works.^15,29^ The dataset is chemically diverse, spanning several reaction classes, reflected in the large span of the target property in Fig. 2.

Correspondingly, no “direct” model (without pre-training on lower levels of theory)^13–15^ has predictive mean absolute errors (MAEs) of less than 4.1 kcal mol^−1^,^31^ (reported error on a single 80/10/10% split). Errors of 4–5 kcal mol^−1^ are in the realm of DFT errors with respect to the CCSD(T) data,^123^ making these predictions as useful as DFT.

#### 2.3.2 Cyclo-23-TS

The Cyclo-23-TS dataset^51^ encompasses 5269 reaction profiles for un-catalyzed [3 + 2] cycloaddition reactions with activation free energy barriers (Δ*G*^‡^) computed at the B3LYP-D3(BJ)/def2-TZVP//B3LYP-D3(BJ)/def2-SVP level. These molecules have a maximum of 50 heavy atoms and a maximum of 94 atoms. The input structures to the ML models are optimized reactants (starting materials) and products. Since the dataset focuses on a single reaction class, the spread in the target property is narrower than for the GDB7-22-TS, illustrated in Fig. 2. The best published MAE is 2.3 kcal mol^−1^,^107^ (reported error averaged over 10 random 90/5/5% splits).

#### 2.3.3 Proparg-21-TS

The Proparg-21-TS dataset^28,124^ contains 754 structures of intermediates before and after the enantioselective transition state of the propargylation of benzaldehyde, with activation energies (Δ*E*^‡^) computed at the B97D/TZV(2p,2d) level. These molecules have a maximum of 52 heavy atoms and a maximum of 89 atoms. The input structures to the ML models are optimized intermediates preceding and following the TS (see Fig. S1†). This dataset is separate from the other two: while it reports energy barriers, these are then converted into enantioselectivity (e.e.) values using the competing barriers of (*R*)- and (*S*)-enantiomers of the product. Thus the focus is not on different reaction classes and transformations, but rather on a stereochemical level, given that stereoisomers of intermediates/TSs are present in the dataset. Correspondingly, there is a narrower spread in the target value. In addition, the dataset is smaller than the other two, providing a more challenging test case for deep learning models. The best reported MAE is 0.27 kcal mol^−1^,^107^ (reported error averaged over 10 random 90/5/5% splits). As the relationship between the barrier and computed selectivity is exponential, a low error in Δ*E*^‡^ is essential.

Unlike the other datasets which have both xyz files containing Cartesian coordinates of reactants and products and (atom-mapped) SMILES strings, this dataset contains only xyz files. xyz were converted to SMILES using the xyz2mol^152^ function of cell2mol.^153^ We note that the original xyz2mol^152^ fails to convert these structures to SMILES, since the encoded chemical rules do not include the elements present in the Proparg-21-TS dataset. cell2mol^153^ extends the program to inorganic chemistry and is able to convert all but one structure to SMILES (noted in the ESI†). On inspection however the resulting SMILES strings are unreasonable, breaking the aromaticity in the cycles.

To address this problem, we generated an alternative set of SMILES strings. The ligands in the intermediates' structures were constructed from a library of fragments.^28^ This allowed for the generation of chemically-meaning SMILES using simple combinatorial rules. We also extend these SMILES strings to partially encode the relevant stereochemistry. Since we noticed no difference in performance of the SMILES-based models depending on the quality of the SMILES strings, we use the lower-quality (generated from cell2mol) and put the results associated with the higher-quality variations, as well as more details as to their construction, in the ESI.†

In order to atom-map the SMILES strings, we modified cell2mol's xyz2mol to keep atom indices from xyz. Since each reaction consists of only one reactant and one product, whose atom order in the xyz files is preserved, the reaction SMILES are easily correctly atom-mapped.

#### 2.3.4 Geometries

The aforementioned datasets provide geometries optimized using DFT. A set of geometries at GFN2-*x*TB^154^ level is taken from ref. 107 for all datasets to compare the performance of the 3D-structure based models with lower quality geometries. Note that for a handful of reactions in the GDB7-22-TS (171) and Cyclo-23-TS (60) sets, at least one of the molecules in the reaction did not converge and therefore are excluded from the geometry quality tests (Fig. 4).

## 3 Results and discussion

**Fig. 3:**
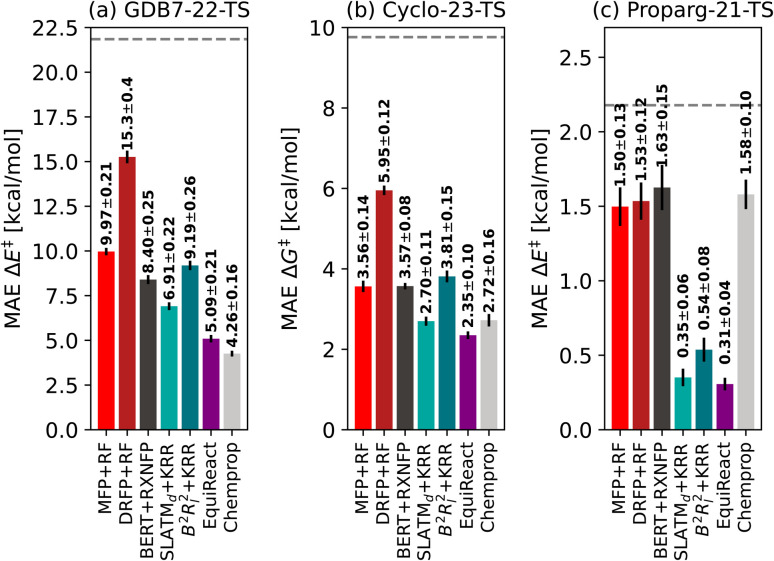
Mean absolute errors of different fingerprints based on 2D structure (MFP + RF, DRFP + RF), language model BERT + RXNFP, 3D-structure based models (SLATM_*d*_ + KRR, *B*^2^*R*_*l*_^2^ + KRR, EquiReact) and 2D graph-based model Chemprop on three different datasets of reaction barriers GDB7-22-TS, Cyclo-23-TS and Proparg-21-TS. The standard deviation of each dataset is given as a dashed line.

Fig. 3 illustrates the performance of various models to predict barriers of 3 diverse datasets: (a) the GDB7-22-TS, (b) the Cyclo-23-TS and (c) the Proparg-21-TS. The three datasets pose different challenges: the GDB7-22-TS set has the largest chemical diversity (and therefore spread in its target property), resulting in a higher overall predicted MAE as well as a larger difference in the performance of various models. This dataset provides a challenging test case for ML models for barrier prediction.

The Proparg-21-TS set provides a different sort of challenge: for a fixed set of starting materials, catalysts with different stereochemistries lead to either the (*R*)- or (*S*)-enantiomeric products. While we constructed modified SMILES strings that enumerate different octahedral arrangements of the ligands (*i.e.*, stereochemistry), they yield the same results as the SMILES strings (see ESI†). Only the 3D-structure-based models, capturing the stereochemistry of the intermediates before and after the enantiodetermining TS, are injective and therefore effective representations. The resulting MAEs are over 20× lower than those of the 2D-structure based representations. The Proparg-21-TS set serves as an important reminder of the diversity of chemical reactions beyond changes in connectivity in reactants and products, by including changes in stereochemistry. Only recently are datasets emerging that explore variations beyond connectivity changes, such as conformations in the recent work of Zhao *et al.*^50^

The Cyclo-23-TS dataset, with a fixed reaction class and no diversity in 3D structure, is the simplest of the three, though distinctions can still be seen between different models, and interestingly, the hierarchy of models for the GDB7-22-TS set are mostly maintained in the Cyclo-23-TS. The following subsections discuss the relative performance of the different models as interesting case studies for ML models for activation barrier prediction.

### 3.1 Models based on three-dimensional geometries

van Gerwen *et al.*^29^ previously compared the performance of various physics-based molecular fingerprints^86,87,89–91^ adapted for the prediction of reaction properties on four different datasets (the GDB7-20-TS,^48^ Hydroform-22-TS,^29^ SN2-20,^29,49^ and Proparg-21-TS^29,124^). The authors found that the SLATM_*d*_ representation resulted in the lowest MAE across the datasets studied. They introduced a related reaction fingerprint, the *B*^2^*R*_*l*_^2^, based on similar design principles, but at a compact size, resulting in a small increase in error compared to SLATM_*d*_. Here, we observe a bigger gap between the performance of the two representations. While *B*^2^*R*_*l*_^2^ + KRR still produces reasonable errors, it becomes less competitive in comparison to other ML models studied here. In line with previously published results,^107^ we find that the more sophisticated architecture in EquiReact (including an end-to-end learned representation and incorporating equivariance for molecular components) allows for improved accuracy compared to the fingerprint models.

**Fig. 4:**
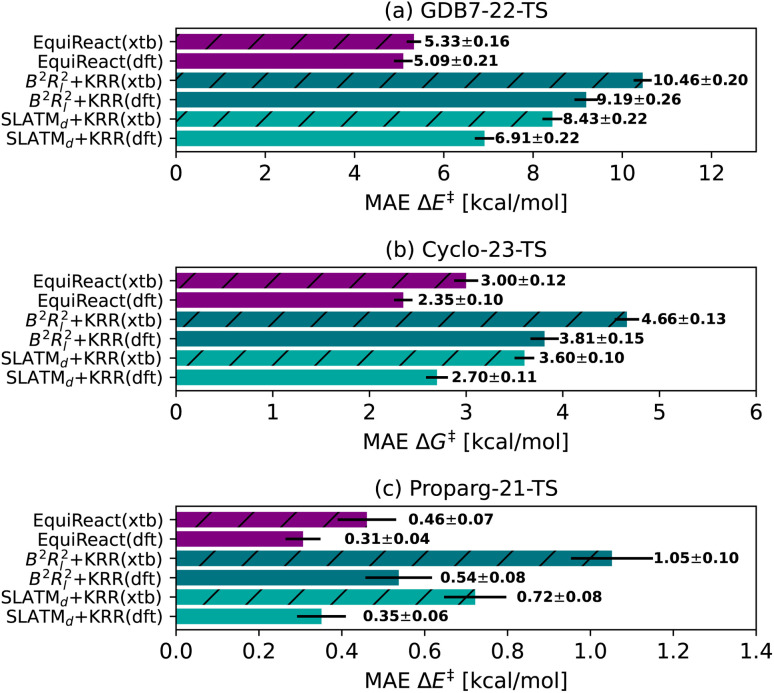
Mean Absolute Error (MAE) for models based on 3D geometry: SLATM_*d*_ + KRR, *B*^2^*R*_*l*_^2^ + KRR and EquiReact using GFN2-*x*TB^154^ (*x*tb) or DFT (dft) levels of theory (ωB97X-D3/def2-TZVP level for GDB7-22-TS, B3LYP-D3(BJ)/def2-SVP for Cyclo-23-TS, B97D/TZV(2p,2d) for the Proparg-21-TS). Note that EquiReact uses a smaller subset of geometries due to technical reasons.^107^ This results in a larger data loss from DFT to *x*TB geometries.

In an out-of-sample setting, optimizing geometries at a high level of theory (here, ωB97X-D3/def2-TZVP level for GDB7-22-TS, B3LYP-D3(BJ)/def2-SVP for Cyclo-23-TS, B97D/TZV(2p,2d) for the Proparg-21-TS) is computationally demanding. Fig. 4 illustrates the resultant MAE with training and predicting on cheaper GFN2-*x*TB geometries. Except for EquiReact on the GDB7-22-TS set, all models suffer from a deterioration of predicted MAE when moving from DFT to *x*TB geometries. The effect is most pronounced for the Proparg-21-TS set, likely because GFN2-*x*TB is not parameterised on 5- or 6-coordinated silicon systems.^154^ The thermochemistry-inspired representations combined with kernel models are more sensitive than EquiReact to the geometry quality, as discussed in ref. 107. SLATM_*d*_ + KRR is also more sensitive than *B*^2^*R*_*l*_^2^ to the geometry quality, since *B*^2^*R*^2^ uses only distances whereas SLATM also uses angles between atoms.

Comparing the models with GFN2-*x*TB geometries to other methods tested, Chemprop and BERT + RXNFP become more attractive options (for Chemprop this relies on atom-mapping quality however, *vide infra*). 3D-structure-based models using xTB geometries still outperform 2D-structure based methods for the Proparg-21-TS set, however, due to the inability of SMILES strings to capture stereochemistry. Methods based on 3D structure remain the only viable option for accurate predictions of datasets with stereochemical diversity.

### 3.2 Graph-based models and their reliance on atom-mapping

The graph-based Chemprop model gives excellent predictions of reaction barriers for the GDB7-22-TS and Cyclo-23-TS datasets. Our final Chemprop MAEs are slightly higher than those published by Heid *et al.*,^31^ because we optimized hyperparameters only on the first fold. For the Cyclo-23-TS dataset, we note that many models (Chemprop, SLATM_*d*_ + KRR, EquiReact) are more accurate than the QM-GNN model published by Stuyver *et al.*^79^ following the publication of the dataset^51^ with a published MAE of 2.96 kcal mol^−1^ (reported error on a single 90/5/5% split, for a non-ensembled model. This is the closest setting to ours published by the authors, otherwise using ensembled models).

In line with Spiekermann *et al.*,^14^ we were surprised to see that the Chemprop model based on 2D graphs of reactants and products could compete with models with 3D information. However, the graph-based models do encode additional information compared to all others considered here: they rely on atom-mapped SMILES as input. The atom-mapped reaction SMILES are used to construct a single graph for a reaction. Then, each node in the graph describes a transformation taking part at each of the atoms involved in the reaction. This is valuable information that resembles a reaction mechanism. It is likely the Chemprop model is more effective than the WLN-type^78–80^ at exploiting the atom-mapping information, as the WLN-type does not explicitly create node features that encode transformations from reactants to products like Chemprop. If the Chemprop model were enhanced with quantum-chemical features, we might see further performance improvements. In any case, high quality atom mapping is an unrealistic pre-requisite for most reaction prediction scenarios (*vide supra*).

**Fig. 5:**
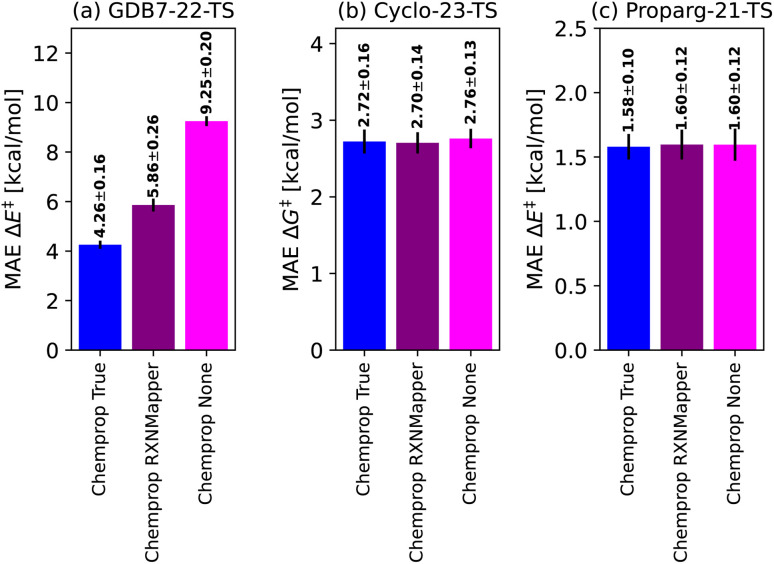
Comparison of the Chemprop model performance depending on the atom-mapping quality: by-hand (“True”), using automatic tools^129^ (“RXNMapper”) or no maps (“None”).

To place Chemprop's performance on fairer footing, we compared its out-of-sample MAE when using high-quality atom-mapping (done by hand or using closed-source software,^155^ “Chemprop True”) to atom-mapping performed by open-source software (“Chemprop RXNMapper”) to the naive graph model without atom-mapping information (“Chemprop None”). Results are illustrated in Fig. 5. The GDB7-22-TS dataset contains exotic chemistry, having been generated using automated PES exploration. This illustrates a realistic test case where RXNMapper might be needed, as mapping by hand or using heuristic rules would be a challenge. Due to the presence of unseen chemistries, RXNMapper struggles to correctly map all the reactions, and correspondingly Chemprop RXNMapper reduces in accuracy compared to Chemprop True, illustrated in Fig. 5a. Entirely removing atom-mapping information results in a poor Chemprop None model.

The RXNMapper is trained on patent data, likely including cycloaddition reactions, therefore predicting the correct maps and Chemprop RXNMapper achieving the same results as Chemprop True for the Cyclo-23-TS dataset (Fig. 5b). For these reactions, atom mapping does not seem critical to good performance, as Chemprop None obtains similar performance to Chemprop True/RXNMapper. Since these reactions consist of a fixed reaction class, atom-mapping likely does not provide new information to the models. Finally, as illustrated in Fig. 5c for the Proparg-21-TS set, all the available Chemprop versions perform poorly, due to graph-based models' inability to distinguish stereochemical variations in the dataset.

These results illustrate that graph-based models are not necessarily best-performing because of the nature of the GNN architecture, but rather because of their dependence on atom-mapping, an additional input that may be challenging to come by for realistic reaction settings. For the GDB7-22-TS dataset, all models without atom-mapping information result in relative high predictive errors (>5.9 kcal mol^−1^), highlighting that more work needs to be done on such challenging sets before ML models can reliably replace computation of activation barriers.

### 3.3 Text-based models

Probst *et al.*^122^ had observed that simple 2D fingerprints (*i.e.*, the DRFP) perform as well as the deep-learned RXNFP^133^ for the tasks of reaction classification on the USPTO 1k TPL dataset^56^ and reaction yield prediction on high-throughput datasets of Buchwald–Hartwig cross-coupling reactions^58^ and Suzuki–Miyaura reactions,^156^ as well as from patents.^47,109^ Jorner and co-workers^54^ previously observed that BERT + RXNFP fingerprints outperformed MFPs in the prediction of experimental activation energies (reaction rates). Now comparing MFPs and DRFPs to deep learned representations built from SMILES for the prediction of computed reaction barriers for the first time, the BERT + RXNFP model outperforms the simpler representations (as well as other sophisticated representations/models). The BERT + RXNFP performs better or equivalently to *B*^2^*R*_*l*_^2^, within standard deviations, on the GDB7-22-TS and Cyclo-23-TS datasets. Thus, it is feasible to obtain good predictive accuracy of reaction barriers using only (un-mapped) reaction SMILES as input. Since RXNMapper results from an attention head of a related transformer model,^129^ it is possible that a similar unsupervised atom-mapping-like task was performed in its training stages.

These results suggest that a new generation of ML models might be able to achieve accurate predictions on reaction property prediction tasks with less information. It could be interesting to investigate whether a tokenization involving atom-maps could further improve these models.

## 4 Timings and representation sizes

Table 1 gives the training and inference times for a subset of 750 points (80/10/10 split) for each model and dataset combination, as well as a description of the scaling of the dimensionality of the representation with the number of unique elements.

**Table 1:** Training times (including generation of fingerprints/representations) and inference times, for a total dataset size of 750 (split into 80%/10%/10%), trained with previously established optimal hyperparameters. Timings reported for a single random fold. Fingerprint models are run on a CPU, neural networks on a GPU. The representation (rep.) size describes the scaling of the representation size with the number of unique elements *n*

Model	Dataset	Train time (s)	Inference time (s)	Rep. size
DRFP + RF	GDB7-22-TS	4.560	0.005	*O*(1)
	Cyclo-23-TS	7.590	0.008	*O*(1)
	Proparg-21-TS	10.597	0.001	*O*(1)
MFP + RF	GDB7-22-TS	0.855	0.010	*O*(1)
	Cyclo-23-TS	0.672	0.007	*O*(1)
	Proparg-21-TS	0.654	0.008	*O*(1)
*B* ^2^ *R*_*l*_^2^ + KRR	GDB7-22-TS	9.5082	0.0004	*O*(*n*)
	Cyclo-23-TS	41.543	0.001	*O*(*n*)
	Proparg-21-TS	125.3702	0.0006	*O*(*n*)
SLATM_*d*_ + KRR	GDB7-22-TS	1.1902	0.0007	O(*n*^3^)
	Cyclo-23-TS	7.2619	0.0007	O(*n*^3^)
	Proparg-21-TS	13.548	0.0009	O(*n*^3^)
EquiReact	GDB7-22-TS	794.265	0.141	*O*(1)
	Cyclo-23-TS	1171.083	0.538	*O*(1)
	Proparg-21-TS	3735.803	0.207	*O*(1)
Chemprop	GDB7-22-TS	131.331	0.102	*O*(1)
	Cyclo-23-TS	181.070	0.160	*O*(1)
	Proparg-21-TS	507.706	0.197	*O*(1)
BERT + RXNFP	GDB7-22-TS	82.681	2.675	*O*(1)
	Cyclo-23-TS	85.931	2.915	*O*(1)
	Proparg-21-TS	88.639	3.385	*O*(1)

All fingerprint-based models (MFP + RF, DRFP + RF, *B*^2^*R*_*l*_^2^ + KRR, SLATM_*d*_ + KRR) were run on a CPU: an Apple Macbook Pro 2022 with an Apple M2 chip (8 CPU cores, 3.5 GHz). All neural networks (EquiReact, Chemprop, BERT + RXNFP) were run on a GPU-enabled cluster: specifically, an Intel Xeon-Gold based cluster (20 CPU cores, 2.1 GHz) with an NVIDIA V100 PCIe 32 GB GPU. A single CPU is used for all jobs.

Of the 2D fingerprints, MFP is more efficient to train than DRFP by several orders of magnitude. Similarly for SLATM_*d*_*vs. B*^2^*R*_*l*_^2^. In both cases this is due to the former representations being implemented in low-level languages: MFP in C and SLATM_*d*_ in Fortran. *B*^2^*R*^2^ is simpler than SLATM_*d*_, using only two-body terms (as indicated in the scaling of the dimensionality of the representations), and therefore could run faster than SLATM_*d*_. A more efficient implementation of *B*^2^*R*^2^ will soon be released as part of the Q-stack python package.^157^ Only SLATM_*d*_ and *B*^2^*R*^2^ suffer from an increasing representation size with the number of unique elements in the dataset. All other methods fix the size of the representation *a priori*. SLATM_*d*_'s cubic scaling can pose memory errors if working with datasets of diverse molecules.

Molecule sizes increase from GDB7-22-TS (up to 7 heavy atoms) to Cyclo-23-TS (up to 50 heavy atoms) to Proparg-21-TS (up to 52 heavy atoms). While the Cyclo-23-TS and Proparg-21-TS have a similar maximum molecule size, all of the molecules in the Proparg-21-TS set are large, whereas the Cyclo-23-TS set also contains small molecules. The train times therefore increase accordingly from GDB7-22-TS to Cyclo-23-TS to Proparg-21-TS for all methods except the MFP + RF. Since the MFP and BERT + RXNFP operate on SMILES strings, while the SMILES lengths do increase with molecule size, the effect is not as dramatic as the graph-based methods or atom-in-molecule based methods. The DRFP also operates on SMILES strings, but creates a set of sub-structures, where these sets increase in size with increased lengths of SMILES strings.

All deep learning models are more costly to train and use for inference than the fingerprint models. EquiReact, which uses tensor operations, is the most expensive to run. BERT + RXNFP is the most expensive to use for inference due to the data augmentation pre-processing step, but is the cheapest of the deep learning models to train.

In summary, if users have limited compute time and/or no GPU, the fingerprint-based models are the best choice. Based on current implementations, cheaper fingerprint-based models are the MFP + RF and SLATM_*d*_ + KRR respectively for 2D and 3D. SLATM_*d*_ can pose memory problems due to its cubic scaling of representation size with maximum number of elements. BERT + RXNFP is the cheapest deep learning model.

## 5 Recommendations for activation barrier prediction

Following the analysis on the three datasets studied here, we give our general recommendations as to which currently available ML methods might be best suited in different scenarios.

• In the case of **chemically diverse datasets**, such as the GDB7-22-TS,^123^ its precursor the GDB7-20-TS,^48^ and the more recent RGD1 dataset^50^ (in our terminology would be the RGD1-23-TS), where reactions are already mapped or can be readily mapped by RXNMapper,^129^**Chemprop**^13^ offers a promising model, as it can better distinguish between different reaction classes in the dataset.

• In the case of **geometry-sensitive datasets**, such as the Proparg-21-TS,^28,124^ a subset of the RGD1 dataset, and any datasets of enantioselectivity data, models based on **3D geometry**,^29^**especially EquiReact**^107^ offers the best performance. The accuracy of the final model might depends on the **quality of the three-dimensional geometries** of reactants and products provided, depending on how well GFN2-*x*TB captures the systems studied.

• In the case of **minimal input information**: no atom-mapping, nor three-dimensional geometries, **only SMILES strings of reactants and products**, language model **BERT + RXNFP**^56^ offers the best performance. This might be practical for more challenging reactions, where atom-mapping with open-source tools remains a challenge (due to their difference from the data used for pre-training RXNMapper) and estimating three-dimensional geometries is difficult with cheap methods.

• If **resources are limited**, particularly if users do not have access to a GPU, the fingerprint models are the best choice. Based on current implementations, the cheapest models are the **MFP + RF**^116^ and **SLATM**_***d***_**+ KRR**^29^ for 2D and 3D models respectively. If users are working with diverse datasets, *B*^2^*R*_*l*_^2^ has a more favourable scaling of representation size with number of elements. **BERT + RXNFP** is the cheapest deep learning model to train (though more expensive at inference time than the other models).

## 6 Conclusion

With the surge in interest in both the dedicated prediction of activation barriers as well as measured performance metrics (yield, enantioselectivity, *etc.*) of chemical reactions, various approaches have emerged to featurize chemical reactions for use in ML models. We compared a diverse set of fingerprints (the MFP and DRFP built from 2D structure, the RXNFP deriving from a pre-trained BERT model on reaction SMILES, the 2D graph-based Chemprop, and 3D-structure based SLATM_*d*_, *B*^2^*R*_*l*_^2^ and EquiReact) on three different datasets of reaction barriers, from the chemically diverse GDB7-22-TS to the fixed reaction class Cyclo-23-TS to the stereochemistry-sensitive Proparg-21-TS. We find that 3D-structure based models are needed particularly for configuration–sensitive reaction properties. The graph-based Chemprop model exhibits excellent performance in the absence of stereochemical diversity, but this is contingent on high-quality atom-mapped reaction SMILES. Language-based models offer the convenience of only using unmapped reaction SMILES as input. These results suggest the way forward for a new generation of ML models for chemical reactions.

## Data availability

The code to reproduce all results can be found at https://github.com/lcmd-epfl/benchmark-barrier-learning. This includes scripts to run the ML models, to parse the results and to generate the plots in the paper. The outputs of the models are saved in the github repository, such that the results can be easily parsed to re-generate the results in the paper. A detailed description can be found in the README of the repository. Three datasets are studied in this work: the Proparg-21-TS, Cyclo-23-TS and GDB7-22-TS. While all sets were previously published and made available open-source, we made some modifications to these sets and made these versions available in two places: in the same github repository mentioned above, as well as on zenodo at the following link: https://zenodo.org/record/8309465. This record includes the datasets studied as well as the saved ML models.

## Author contributions

The project was conceptualized by P. v. G. and C. C. M. F. and Y. C. A. contributed to preliminary experiments initiating this work. Data was curated by P. v. G. and K. R. B. Data was analyzed by P. v. G., K. R. B. and Y. C. A. The original draft was written by P. v. G. with reviews and edits from all authors. C. C. provided supervision throughout and is acknowledged for acquiring funding.

## Conflicts of interest

There are no conflicts to declare.

## Supplementary Material

DD-003-D3DD00175J-s001
