# Supplementary material for: Benchmarking machine-readable vectors of chemical reactions on computed activation barriers
Source: Digit Discov. 2024 Mar 7;3(5):932–43. doi: 10.1039/d3dd00175j (PMC11094696; doi:10.1039/d3dd00175j)
Supplement: DD-003-D3DD00175J-s001 [file DD-003-D3DD00175J-s001.pdf]

Electronic Supplementary Material (ESI) for Digital Discovery.
This journal is © The Royal Society of Chemistry 2024

Supplementary Material for "Benchmarking machine-readable vectors of chemical reactions on computed activation barriers"

Puck van Gerwen, Ksenia R. Briling,
Yannick Calvino Alonso, Malte Franke
and Clemence Corminboeuf*

Laboratory for Computational Molecular Design,
Institute of Chemical Sciences and Engineering,
Ecole Polytechnique Federale de Lausanne,
1015 Lausanne, Switzerland

March 8, 2024

Contents

S1 Hyperparameters **S2**
S1.1 Kernel models S2
S1.2 Random Forest models S2
S1.3 Chemprop S3
S1.4 Language models S3
S1.5 EquiReact S3

S2 Data augmentation for language models **S4**

S3 RXNMapper confidence **S4**

S4 SMILES for Proparg-21-TS **S4**
S4.1 Failed conversion S4
S4.2 Comparison of `xyz2mol`, fragment-based and stereochemistry-enriched SMILES S4

*email: clemence.corminboeuf@epfl.ch

S1 Hyperparameters

S1.1 Kernel models

The best hyperparameters were found in a grid search of kernel function $\in$ [Laplacian (`laplacian`) $K_\mathrm{L}(\mathbf{x},\mathbf{x}') = \exp\left(-\gamma||\mathbf{x}-\mathbf{x}'||_1\right)$, Gaussian (`rbf`) $K_\mathrm{G}(\mathbf{x},\mathbf{x}') = \exp(-||\mathbf{x}-\mathbf{x}'||_2/(2\sigma^2))$]; the Laplacian kernel coefficient $\gamma \in [10^{-5}, 10^{-4}, 10^{-3}, 10^{-2}, 0.1, 1]$; Gaussian width $\sigma \in [1, 10, 100, 10^3, 10^4]$; and L2-regularization parameter $\lambda \in [10^{-10}, 10^{-7}, 10^{-4}]$. The best hyperparameters for the first fold are given for the two representations SLATM$_d$ and $B^2R^2_l$ in Table S1.

Dataset (geometries)	SLATM$_d$			$B^2R^2_l$		
	kernel	γ / σ	λ	kernel	γ / σ	λ
GDB7-22-TS (DFT)	`laplacian`	10^{-2}	10^{-10}	`laplacian`	10^{-4}	10^{-4}
GDB7-22-TS (xTB)	`laplacian`	10^{-2}	10^{-4}	`laplacian`	10^{-4}	10^{-4}
Cyclo-23-TS (DFT)	`laplacian`	10^{-3}	10^{-10}	`laplacian`	10^{-4}	10^{-10}
Cyclo-23-TS (xTB)	`rbf`	10^{1}	10^{-10}	`rbf`	10^{2}	10^{-4}
Proparg-21-TS (DFT)	`rbf`	10^{4}	10^{-10}	`laplacian`	10^{-5}	10^{-10}
Proparg-21-TS (xTB)	`rbf`	10^{3}	10^{-4}	`rbf`	10^{3}	10^{-4}

Table S1: Hyperparameters for the SLATM$_d$ and $B^2R^2_l$ representations combined with kernel models, optimized on the first fold.

Note that in earlier works,[S1,S2] only Gaussian kernels were considered for these representations. The inclusion of Laplacian kernels in the hyperparameter optimization improved the accuracies of the ML models for most of the datasets studied here.

S1.2 Random Forest models

The best hyperparameters are found in a Bayesian search through the parameter space detailed in Table S2. The best parameters found for the DRFP and MFP models are given in Table S3.

Parameter	Search space
`max_depth`	[10, 20, 30, 40, 50, 60, 70, 80, 90, 100]
`n_estimators`	[100, 155, 211, 266, 377, 433, 488, 544, 600]
`max_features`	[`log2`, `sqrt`]
`min_samples_split`	[2, 5, 10]
`min_samples_leaf`	[1, 2, 4]
`bootstrap`	[`True`, `False`]

Table S2: Search space for the Bayesian optimization of hyperparameters for RF models.

Parameter	MFP			DRFP		
	GDB7-22-TS	Cyclo-23-TS	Proparg-21-TS	GDB7-22-TS	Cyclo-23-TS	Proparg-21-TS
`max_depth`	90	50	10	70	80	10
`n_estimators`	355	233	477	172	294	50
`max_features`	`sqrt`	`sqrt`	`sqrt`	`sqrt`	`sqrt`	`sqrt`
`min_samples_split`	5	2	5	2	2	5
`min_samples_leaf`	1	1	1	1	1	1
`bootstrap`	`False`	`False`	`False`	`False`	`False`	`False`

Table S3: Optimal parameters obtained from a hyperparameter search for the MFP and DRFP representations used with RF models, for each dataset.

S1.3 Chemprop

The hyperparameter space to be searched is as implemented in `chemprop`[S3] version 1.6.1, and summarized again in Table S4.

Parameter	Search space
`ffn_hidden_size`	[300, 400, 500, 600, 700, ..., 2400]
`depth`	[2, 3, 4, 5, 6]
`dropout`	[0.0, 0.05, 0.1, ..., 0.4]
`ffn_num_layers`	[1, 2, 3]

Table S4: Search space for the Bayesian optimization of hyperparameters for the CHEMPROP model.

The best parameters resulting from the search are summarized in Table S5.

Parameter	GDB7-22-TS	Cyclo-23-TS	Proparg-21-TS
`ffn_hidden_size`	2100	1500	400
`depth`	5	6	5
`dropout`	0.15	0.05	0.25
`ffn_num_layers`	2	3	3

Table S5: Best parameters resulting from the hyperparameter search for each dataset for the CHEMPROP model.

S1.4 Language models

Hyperparameters were optimized on the first fold of the datasets, without data augmentation. The learning rate `lr` and dropout probability p were optimized over a grid: $\texttt{lr} \in [10^{-5}, 5\times10^{-5}, 10^{-4}, 5\times10^{-4}, 10^{-3}]$, $p \in [0.2, 0.4, 0.6, 0.8]$. The best parameters are summarized in Table S6.

Parameter	GDB7-22-TS	Cyclo-23-TS	Proparg-21-TS
`lr`	10^{-4}	10^{-4}	5×10^{-4}
p	0.2	0.2	0.2

Table S6: Best parameters resulting from the hyperparameter search for each dataset for the language models.

S1.5 EquiReact

The optimal parameters for the EQUIREACT models are taken from Ref. S4 and are repeated below.

Parameter	GDB7-22-TS	Cyclo-23-TS	Proparg-21-TS
atom mapping mode	True	None	None
n_s	64	64	64
n_v	64	48	16
n_g	48	48	16
n_{conv}	3	3	3
$r_{\max}$, Å	2.5	2.5	5
n_{neigh}	50	10	50
p_d	0.05	0.05	0.1
`sum_mode`	`node`	`both`	`node`
`combine_mode`	`diff`	`mlp`	`diff`
`graph_mode`	`vector`	`energy`	`vector`
learning rate	10^{-3}	5×10^{-4}	10^{-3}
weight decay	10^{-5}	10^{-4}	10^{-5}

Table S7: Best model hyperparameters for EQUIREACT for the three datasets, evaluated on the first set of random splits, as reported in Ref. S4

S2 Data augmentation for language models

To verify whether the inclusion of data augmentation was beneficial, models were tested with 10 SMILES randomizations (rand) and none. No intermediate numbers of randomizations were tested. The optimal set of hyperparameters listed in Table S6 were used. Models were trained with a batch size of 32. The resulting MAEs are summarized in Table S8. Since the models showed either improvement or no change with data augmentation, we used 10x data augmentation for the results in the main text.

Dataset	No rand MAE [kcal/mol]	10 rand MAE [kcal/mol]
GDB7-22-TS	10.70 ± 0.40	8.40 ± 0.25
Cyclo-23-TS	3.81 ± 0.14	3.57 ± 0.08
Proparg-21-TS	1.62 ± 0.15	1.63 ± 0.15

Table S8: A comparison of model MAEs for various datasets with and without data augmentation (10-fold), trained for 1 epoch.

S3 RXNMapper confidence

`RXNMapper` reported an average confidence indicated in Table S9 for each of the three datasets. The confidence is especially low for the Proparg-21-TS dataset, due to the foreign nature of the chemistry compared to the data on which it was pre-trained.

Dataset	Confidence
Cyclo-23-TS	0.64
GDB7-22-TS	0.87
Proparg-21-TS	0.28

Table S9: Average reaction mapping confidence as reported by `RXNMapper`.

S4 SMILES for Proparg-21-TS

S4.1 Failed conversion

The Proparg-21-TS dataset[S5] contains 754 structures of intermediates before (**2**) and after (**3**) the rate-limiting stereocontrolling transition state of the catalytic benzaldehyde propargylation reaction (Fig. S1). For one of the entries in the dataset[S5,S6] (labelled as `3jbp3R`) the **2** structure corresponds to a non-covalent complex between **1** and benzaldehyde. `xyz2mol` from `cell2mol` failed to produce a disconnected molecular graph, thus we excluded this entry from training.

S4.2 Comparison of `xyz2mol`, fragment-based and stereochemistry-enriched SMILES

`xyz2mol` from `cell2mol` correctly determined atom connectivity from xyz but failed in assigning bond types and atom charges. For example, for **2** (Fig. S2a) in entry `1abp1R`,[S5] the SMILES string is

```
[H][C+]([H])C[C+]([H])[Si+2]1(Cl)(Cl)(O[C-]([H])[C-]2[C+]([H])[C+]([H])[C-]([H])[C-]([H])[C-]2[H])ON2[
    C+]([H])[C-]([H])[C-]([H])[C+]([H])[C-]2[c+]2[c+]([H])[c-]([H])[c+]([H])[c-]([H])n2O1
```

which corresponds to an unreasonable structural formula shown in Fig. S2b. To address this issue, we built an alternative set of SMILES strings using dataset-specific knowledge. We will refer to these as "fragment-based" SMILES. They are constructed as follows.

Different entries of the dataset vary by: a) substituents in the bipyridine N,N'-dioxide catalyst; b) ligand rearrangement around the Si center; c) conformation of the coordinated benzaldehyde leading to different enantiomers. Since the catalysts were assembled from a library of fragments[S5,S6] and **2** and **3** core structures persist across the dataset, the SMILES can be constructed using simple combinatorial rules. The resulting SMILES string for **2** of entry `1abp1R` reads

```
[Si-2]1([O+]=[C]([c]2[c]([H])[c]([H])[c]([H])[c]([H])[c]2[H])[H])([C](=[C]=[C]([H])[H])[H])([Cl])([Cl
    ])[O][n+]2[c]([c]([H])[c]([H])[c]([H])[c]2[H])-[c]2[c]([H])[c]([H])[c]([H])[c]([H])[n+]2[O]1
```

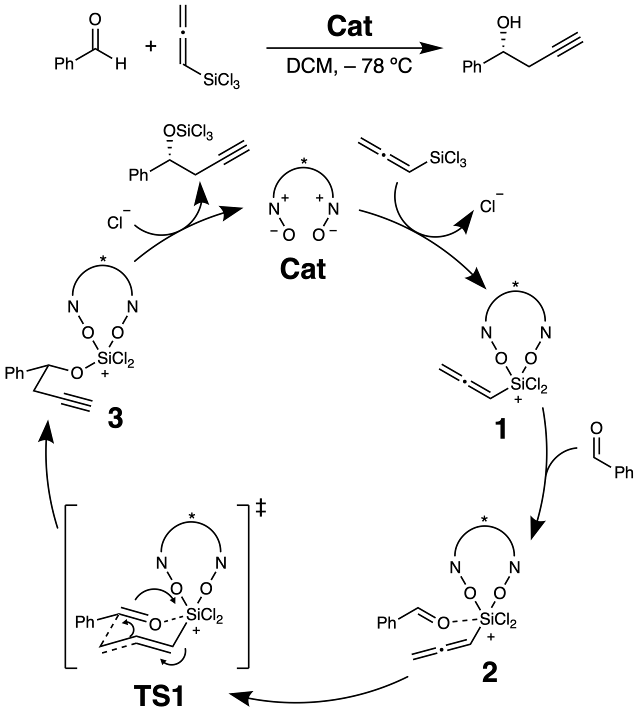

Figure S1: The catalytic cycle for benzaldehyde propargylation reactions (reused with permission from Laplaza *et al.*[S7]).

and the corresponding structural formula is shown in Fig. S2c. For a fraction (∼ 10%) of dataset entries, in **2** and/or **3** the bipyridine N,N'-dioxide ligand forms only one bond with Si leaving it penta- or tetracoordinated, respectively. These cases are automatically detected and alternative SMILES are assigned. These fragment-based SMILES have correct bond types and reasonable formal atom charges.

We also assigned the formal atom charges to correspond to reasonable Lewis structures. The resulting SMILES contain a hypervalent Si atom (as if it was in $SiF_6{}^{2-}$) and `rdkit`[S8] cannot read them with default settings. We suggest to use the following code snippet:

```
from rdkit import Chem
smiles_string = ...
mol = Chem.MolFromSmiles(smiles_string, sanitize=False)
Chem.SanitizeMol(mol, Chem.SanitizeFlags.SANITIZE_ALL ^ Chem.SanitizeFlags.SANITIZE_PROPERTIES)
mol = Chem.RemoveHs(mol, sanitize = False)
Chem.SanitizeMol(mol, Chem.SanitizeFlags.SANITIZE_ALL ^ Chem.SanitizeFlags.SANITIZE_PROPERTIES)
```

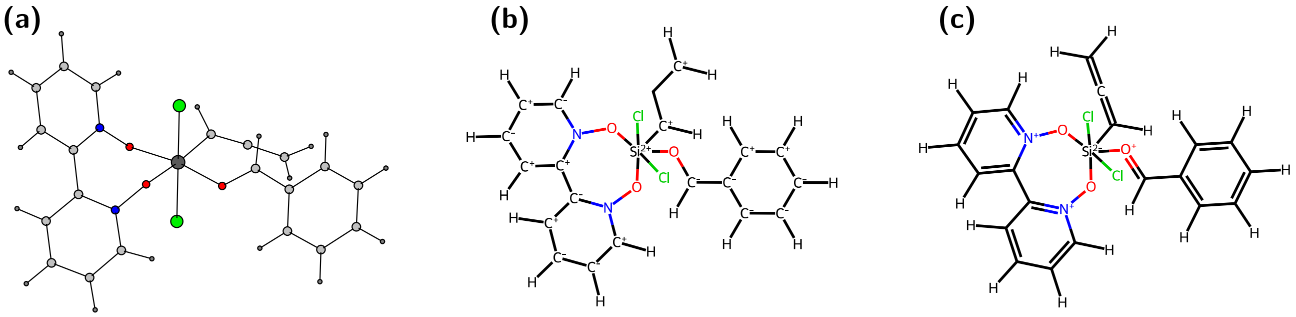

Figure S2: *(a)* 3D structure of **2** of dataset entry `1abp1R` and its structure formulae corresponding to SMILES generated by *(b)* `cell2mol`'s `xyz2mol` and *(c)* using combinatorial rules. In *(b)* the allene atom is mistakenly saturated. Note that in *(c)* the Si stereo configuration is not reflected.

We constructed a third set of SMILES that partially encode stereochemistry information, which we will refer to as "stereochemistry-enriched" SMILES. They are constructed as follows. The ligand rearrangement in **2** is indicated with `@OH1`–`@OH30` SMILES stereo tags. The trigonal bipyramidal configuration of **3**, as well as configurations of "alternatively" coordinated Si atoms in **2** and **3**, are not specified. Even though the coordinated benzaldehyde conformation in **2** cannot be encoded with SMILES, it matches the configuration of the bound

phenyl propargyl ketone product in **3** which is indicated with `@` and `@@` tags. This resulted in a set of injective SMILES for the Proparg-21-TS dataset. We note that the procedure we used to atom-map the SMILES (graph matching with the atom-mapped graphs obtained from xyz) could have switched the two Cl atoms possibly affecting the CHEMPROP results.

Performance of the 2D-based methods with the three types of SMILES strings (from xyz2mol, fragment-based and stereochemistry-enriched) is compared in Table S10. While the SMILES quality improves from xyz2mol SMILES to combinatorial, only the MFP benefits slightly from the change. Most methods do not change. Including stereochemistry information again leads to a marginal improvement in most cases, notably in the CHEMPROP, but actually reduces the ability of other models including the DRFP. Unfortunately, the SMILES-based methods are not written to exploit stereochemistry information. The DRFP for example looks for circular substructures in reactants and products. The presence of stereochemistry flags may confuse the notion of these substructures.

Model	SMILES source		
	`xyz2mol`	combinatorial	fixarom
MFP+RF	1.50 ± 0.13	1.46 ± 0.12	1.58 ± 0.17
DRFP+RF	1.53 ± 0.12	1.51 ± 0.12	1.45 ± 0.12
BERT+RXNFP	1.62 ± 0.15	1.55 ± 0.13	1.61 ± 0.16
CHEMPROP True	1.58 ± 0.10	1.59 ± 0.12	1.55 ± 0.10
CHEMPROP RXNMapper	1.60 ± 0.12	—	—
CHEMPROP None	1.60 ± 0.12	1.59 ± 0.13	1.55 ± 0.13

Table S10: Comparison of 2D models MAEs [kcal/mol] for the Proparg-21-TS dataset on different sets of SMILES. The BERT+RXNFP results are given for datasets without data augmentation, 10-fold cross-validated, run for 5 epochs. The hyperparameters are those for the `xyz2mol` SMILES strings.

These results point to weaknesses in current 2D-structure based methods to handle datasets that vary in stereochemistry, even when the stereochemistry is explicitly encoded in the SMILES strings.

References

[S1] P. van Gerwen, A. Fabrizio, M. Wodrich and C. Corminboeuf, *Mach. Learn.: Sci. Technol.*, 2022, **3**, 045005.

[S2] P. van Gerwen, M. D. Wodrich, R. Laplaza and C. Corminboeuf, *Mach. Learn.: Sci. Technol.*, 2023, **4**, 048002.

[S3] K. Swanson, K. Yang, W. Jin, L. Hirschfeld and A. Tam, *chemprop*, `https://github.com/chemprop/chemprop`, 2022.

[S4] P. van Gerwen, K. R. Briling, C. Bunne, V. R. Somnath, R. Laplaza, A. Krause and C. Corminboeuf, *arXiv preprint*, 2023, arXiv:2312.08307.

[S5] S. Gallarati, R. Fabregat, R. Laplaza, S. Bhattacharjee, M. D. Wodrich and C. Corminboeuf, *Chem. Sci.*, 2021, **12**, 6879–6889.

[S6] A. C. Doney, B. J. Rooks, T. Lu and S. E. Wheeler, *ACS Catal.*, 2016, **6**, 7948–7955.

[S7] R. Laplaza, S. Gallarati and C. Corminboeuf, *Chemistry–Methods*, 2022, **2**, e202100107.

[S8] RDKit, *"Open-source cheminformatics"*, 2023, `http://www.rdkit.org`.
